# Mississippi Dental Association

**Published:** 1897-07

**Authors:** J. M. Walker


### Mississippi Dental Association.

Reported for the DENTAL REGISTER by Mrs. J. M. Walker.

The Fourth Annual Session of the Mississippi Dental Association was held in Jackson, Miss., the permanent home of the Association, April 7th, 8th and 9th, 1897.

The meeting was opened with prayer by the Rev. H. M. DuBose, after which the President, Dr. Frank H. Smith, of Water Valley, read his annual address. His first recommendation for the consideration of the association, was, that the Board of Dental Examiners should endeavor to have all applicants show their skill by clinics. In his address the president urged the importance of further efforts toward the extermination of the hordes of quacks and charlatans, outlaws and guerrillas, occupying the intrenchment of ignorance, which bars the advance of progressive minds with the bluster of merits which they do not possess, and the cheapness of work which is dear at any price. How to protect humanity from our proportion of these quacks is fully within the province of the State Association. He also urged the importance of securing exemption from jury duty and privilege-tax. He said: "In their wisdom the legislative power has exempted all those whose duty it is to alleviate the misfortunes and miseries of humanity, from the obligation of jury service, except the dental surgeon. Why he, who is constantly called upon in critical emergencies, should be compelled to lay down his beneficient work to take this public service, we can not see. The burden of a privilege tax now imposed upon a class whose remuneration is not commeasurate with their deserts, and not equal to that of a competent physician, should also be removed." Other recommendations of an equally practical character were urged upon the consideration of the association.

The committee to whom the address was referred reported favorably upon the suggestions of the president, which were discussed at some length, especially the recommendation of clinics by applicants for license. This was considered impracticable by the members of the present board, because of the expense that would be involved in the purchase of chairs, engines, instruments, etc. ; the cost of storage from one meeting to the next, the damage to instruments from lying unused and uncared for, for a year except the few days of the annual meeting of the board, the additional time the members of the board would have to give without compensation, etc. In the course of the discussion it was pointed out that enough second-hand chairs could probably be found among the members, for little if anything more than the cost of transportation, which would also be serviceable for the clinics of the association, which are always made an important feature of the meeting; that the applicants being mostly young graduates just from college would have their own student's outfit, probably including some portable chairs; that the time given to the examinations could not necessarily be very much extended and the examination in theory could be held first, only those who passed in theory being required to clinic to show their skill also. Dr. Joseph Bauer of the Louisiana State Board being present, gave an interesting account of the way in which the Louisiana Board Clinics are conducted, every applicant being required to make a clinic in both operative and prosthetic dentistry, the applicants being conducted in squads to the offices of the different members of the board for this purpose.

At the night session Dr. J. P. Broadstreet, Grenada, read a paper on "Education." Dr. Broadstreet emphasized the point that in order for the dentist to accomplish the greatest good, he must have the hearty co-operation of his constituency, and in order to obtain this co-operation the people must be educated up to the point where they will realize their needs and appreciate the benefits to be derived from a proper knowledge and a judicious care of their masticating organs. He deplored the fact that the children of our country are being allowed to grow up in dense ignorance of the great importance of caring for and preserving the condition of these valuable organs, eliciting no concern whatever on the part of their parents until they feel the pain and suffering; then the offender must promptly be got rid of —*extraction*, is the only remedy in the minds of the public. This is not from lack of solicitude for the welfare of the child but solely from lack of education along this line. The remedy for this state of affairs suggested by Dr. Broadstreet, is, the appointment by the State Dental Associations, of lecturers, whose duty it shall be to visit the schools throughout the State, and lecture in them on this subject, getting both teachers and children interested in the subject. The children would talk about it when they went home, and so we would get the parents interested; in this way all classes in the community would be reached. When this machinery is once put in motion, there is no telling what might not be accomplished.

For the dentist to properly educate his constituency he must himself be properly educated; he must also keep up with the advances made in the profession of dentistry. There should be some way by which every dentist should be compelled to read one or more of the dental journals, and to attend at least one dental association, and in case of failure to do so he should either forfeit his license to practice, or submit to an examination along the line of advanced theories and discoveries.

The teachers in the Public schools (in Mississippi) are required at stated intervals, to be re-examined, to see if they are keeping up with the constant advancement along educational lines. If they fail to come up to the requirements, they are disqualified to teach until they shall have remedied their defects. If such a system as this could be applied to practitioners of dentistry, it would be an advanced step toward elevating the whole profession to a high plane of success and usefullness; a high standard of excellence upon which to base its claim to the respect of the world and the confidence of the people.

The paper was discussed by Profs. Frank Holland and W. E. Walker of the Dental Department, Southern Medical College, Atlanta, Ga., R. B. Freeman and J. A. Dale, Dental Department, Vanderbilt University and Drs. West, L. A. Smith, W. T. Martin, F. H. Smith and others, Dr. Broadstreet closing the discussion.

The second day of the meeting was set aside for clinics, but a storm coming on it grew too dark for the operation in hand. The convention was therefore called to order at 3 p. m., when the report of the committee on the President's address was received, after which Dr. R. K. Luckie presented the Association with a type-written copy of the material which had been collected and formulated into a history of dentistry in Mississippi at the time of the World's Columbian Dental Congress in 1893. This was designed to form a chapter of the History of Dentistry in the United States, to be published under the auspices of the Congress. As this has never been published it was deemed desirable to place the material in the hands of the State Association.

The matter was referred to a committee on whose recommendation it was decided to publish the matter in connection with the proceedings of the present meeting.

At the night session Dr. W. B. Fulton of the Birmingham Dental College read a paper entitled: "To what extent are we justified in giving our patients systemic treatment?"

The author traced the close relations existing between the teeth and the general organism in the relation of cause and effect, showing clearly the inutility of merely local treatment in many cases without systemic treatment for the constitutional dyscrasia traceable as a cause of the local manifestation.

Pyorrhea Alveolaris being one of the diseases touched upon by Dr. Fulton in his paper, before proceeding to its discussion, Dr. Geo. B. Clement, of Macon, read a paper on the etiology of this disease, followed by Dr. T. Stewart of Greenville, on its treatment.

In his paper Dr. Clement further elaborates the theory of which he is the author and which he has demonstrated by the aid of microscopical sections of the cementum of teeth affected by this disease—namely, that from some undiscovered cause, the density of the cementum is carried beyond its physiological condition on hyper-calcic deposits, or infiltration, the result being exfoliation of the tooth, because, by a perverted nutrition, there is an interference with those conditions, by and through which only, is there hope of vital union. This condition of hyper-calcic cementosis, Dr. Clements considers to be the cause of pyorrhea alveolaris.

Dr. Clement in this paper briefly notices the various theories which have been advanced as to the etiology of this disease, and the various methods of treatment dating from Fauchard, 1746, to the Uric acid theory of to-day, and says: "With all this theory of cause and treatment, can we to-day take charge of a genuine, specific case, and cure it? I claim we can not."

Dr. Stewart's paper was devoted more especially to the practical details of treatment of this diseaee, which he does not con sider by any means incurable. He said: "Whatever be the cause, whether local or systemic, whether associated either with salivary or serumal calculus, or with none at all; whether the pockets be deep and easily penetrated, or whether the sensitive gum adhering firmly to the tooth; whether pus be discernable or not; whether the teeth be loose or firm; the treatment is virtually the same, viz: thorough breaking up of diseased tissue and removal of deposits if present, making a stimulating and solvent application, and protecting the pockets (if any), from the ingress of micro-organisms until nature has a chance to establish a healthy condition.

"At the first sitting remove only visible and easily accessible deposits and dismiss the patient for two or three days with the following prescription:

℞ Acidi Tannici, - - - ʒij;

Acidi Carbolici, - - - gttxxx

Glycerinae, - - - - ℥ı

Aquae q s, - - - - ℥√iii m — Sig.

Mouthwash: Three times daily.

This gets things in shape to begin work, the case being supposed to have several teeth seriously affected, calculus abundantly present, the gums highly inflamed and bleeding at every touch at the time of the first sitting, at which time great care must be exercised if you hope to see the patient again.

"At the next sitting inject with hypodermic syringe a small quantity of a 1 percent sol. cocaine. Then with a suitable instrument scrape the cementum thoroughly to the bottom of the pocket."

Now follows what Dr. Stewart considers the most essential part of the treatment: A thin flexible chisel-shaped lancet, three edges cutting on the point and sides, is to be deftly carried toward the apex beyond the bottom of the pocket, and holding it firmly against the side of the root, carry it all the way around the tooth, separating the gum entirely from the tooth.

If no discernable pocket be present, the lancet is just as freely used. This operation, under the influence of cocaine, or eucaine which is preferable, is absolutely painless and gives free acces to the root which is now to be scraped and chiseled heroically all around—not merely to remove the tartar, but to completely tear away the thickened and congested membrane, even if in so doing the cementum itself is removed to a considerable depth. A speedy and permanent cure is impossible unless this is thoroughly done. The overlying gum is also to be thoroughly lacerated, tearing away the inner glazed surface to induce new granulation and a re-union of the gum with the tooth. During this process syringe repeatedly and forcibly with hot water, in which a few crystals of permanganate of potash have been dissolved, using sufficient force to throw out all debris.

A special syringe point is necessary for this, which should be bent at nearly a right angle at a length sufficient to reach the apex of the tooth. From one to two hours time will be necessary for the two teeth to be operated on at one sitting. When the surgical operation has been completed, a solution of permanganate of potash (20 grains to the ounce of water), is injected with Dunn's syringe, followed after a few minutes by a 50 percent solution of the commercial sulphuric acid and introduced on a quill or orange wood stick. This strong solution is used for its solvent action on the cementum itself. The pockets being now protected from saliva, a protective covering is flowed over them, the formula being that of Dr. E. C. Kirk:

| | | |
|---|---|---|
| ℞ | Shellac, - - - | ℥ i |
| | Benzoin, - - - - | ʒ ii |
| | Balsam Tolu, - - | ʒ ii.ss |
| | Carbolic Acid, - - | ʒ ii |
| | Oil Cinnamon, - - | ʒ i ss |
| | Saccharine, - - - | ʒ i.ss |
| | Alcohol q s, | |

Evaporate the alcohol from the application with hot air, leaving a covering that will protect the pockets for several days. The patient is instructed not to use tooth brush or pick and is given the following prescription:

℞ Potassi Permanganate, gr. viij
Aquae, - - - - - ℥ viij

Sig. Rinse the mouth every two hours. This must be faithfully done for weeks and sometimes months. The tannic acid wash prescribed on the first day is also to be continued three times daily, for a two-fold purpose—to relieve inflammation and to constringe the turged gum. Hydronaphol, listerine or pasteurine may be substituted for the old-fashioned (but still good) permanganate wash. This completes the treatment. There is no secondary treatment beyond the faithful use of the prescribed washes. At the end of a month if any tooth is not doing well, simply repeat the operation on that particular tooth. Scraping, or even with the engine burring, away the outside layer of the cementum and again applying the acid and the protective covering. In case of a stubbornly loose tooth, it may sometimes be advisable to devitalize, remove the nerve and ream out the root canal, treating t from the inside as well as from the outside—this only as a last resort, and absolutely not till then—opening with the drill through the foramen until you have a full opening, when syringe out forcibly with hot water and permanganate and pump in a 25 percent solution sulphuric acid through the opening at the apex, first cutting it out around over the root as much as possible and forcing the acid through into the mouth at the gum margin. This reaches the affected portions of the alveolar process itself. Carry into the canal a strand of cotton saturated with a 60 percent sol. sulp. acid and seal it up with a temporary filling, allowing it to remain twenty-four hours. Renew every day for several days. Fill the root and Nature will do the rest.

Admitting that Dr. Clement's theory is the correct one, may it not be possible that the sulphuric acid thus used had some solvent effect on the lime salts in the partially obstructed canaliculi, penetrating them and stimulating to renewed activity the sluggish vitality in the tissues? Certain it is that there is established a connective tissue that is quite firm in its attachment.

Too much stress can not be laid on the importance of thoroughly and deeply scraping the roots and lacerating the tissues. Failure is due only to lack of thoroughness in the surgical operations. The only precaution necessary is not to scrape such furrows and grooves as to leave sharp angles to irritate the overlying gums.

As to fees each tooth should be charged for separately on the basis of the time consumed in the operation and the amount that could have been earned in the same time by other operations in the mouth. On this basis, from 50 cents to $1.50 is by no means exorbitant when from one to twenty-five teeth are involved.

In the discussions of these papers, Dr. Clement condemned Dr. Stewart's heroic treatment as absolutely destroying all vital union, the result being the formation of cicatricial tissue only.

Dr. Holland said that he had never seen a tooth restored to healthy conditions after the apex had been drilled through. The very first step in Dr. Stewart's treatment condemns it in Dr. Holland's opinion, as there can not be union by first intuition after the hypodermic injection of cocaine into the tissues and instead of having lymph you will have a granulated condition. When pyorrhœ destroys the membrane it is by chronic inflammation. Destroying the dental nerve in the treatment of this disease appears a most unreasonable proceedure. There is already an awful condition of the surrounding tissues and a drill through the apex seems egregious—can not conceive of the necessity for for it. The most potent cause of this disease is the introduction into the system of mercury, of which the first indications are found in the mouth—ptyalism—a gingival inflammation, first acute, then chronic pyorrhœ alveolaris; this is one of the special causes of the disease.

Removing the deposits, cleaning thoroughly, followed by an escharotic to eat away the unhealthy granulations may afford some benefit and induce a contraction of the gums, but there will never be any union after the membrane has been destroyed. The disease originates in systemic troubles and must be treated constitutionally--systemically--not merely locally.

Dr. West said that an important point in the discussion of this disease is to have a definite idea as to what constitutes pyorrhœ alveolaris. The conditions are so varying that one man's treatment of what he calls pyorrhea may be successful in his hands, but a total failure in the hands of another from his different view of what pyorrhea is. We have it described as with pus and without pus; with deposits and without deposits, with pockets and without pockets; with loosening and without loosening. I have had cases, and Dr. Walker has described cases, where the disease seemed to originate at the apex of the root and appeared to be working up toward the gum margin, but left the margin absolutely intact; with no opening whatever leading to the affected point. This is what confuses us in the discussion of pyorrhœ alveolaris.

Dr. Nesbit does not think it right to put the disease down as *incurable.* If the disease is taken in time and proper systemic treatment combined with surgical manipulation, the disease can be permanently cured in many cases.

Dr. A. B. Kelly: Even though some cases may be ultimately lost, it is our duty to study the disease thoroughly and that persistently. Even if we do not finally succeed we may prolong the usefulness of the affected teeth. We may give relief even if we do not cure.

Dr. L. N. Smith: The devitalization of the nerve must induce a change in the condition of the surrounding tissues. Nature makes provision for changed conditions that we do not understand. As the result of Dr. Stewart's treatment, nature steps in and does what we can not do.

At the next session, the Committee on Topics and Essayists for the next Annual Meeting, announced the following programme: Suggestion, Essayist Dr. R. R. Freeman; Dental Chemistry, Dr. W. B. Fulton; Riggs' Disease, Drs. Clement and Stewart; Interests of the Dentists of this State, Dr. C. B. Hines; Differential Diagnosis, Dr. Fr. Holland; Cataphoresis, Dr. J. B. Askew.

A communication from Dr. R. Ottolengui in regard to the advisability of a general law regulating medical and dental patents, elicited an interesting discussion, the concensus of opinion being, that the inventor who gives both time and labor, has a right to protection and recompense.

A communication from Dr. Arrington in regard to the proposed union of the American and Southern Dental Associations, was, after some discussion, laid on the table on the ground that the time has not arrived for local action, the matter being yet in the hands of the committee appointed by the two associations. The report of these committees must be heard as a basis for future discussion.

The Committee on Clinics reported some beautiful contour work done by Dr. Frank Holland of Atlanta, and ideal crown work by Dr. J. A. Dale of Nashville.

Dr. T. C. West, of Natchez, exhibited some original methods in plate work, using a vulcanized black rubber base plate, festooning the gums by manipulating moldine under rubber dam stretched over the teeth, warming the teeth and setting them in rubber instead of waxing up; and other valuable original methods by which much time is saved and very perfect results obtained in vulcanite work.

A clinic in cataphoresis, to be given by Dr. Walker, was not given; none of the cavities operated on being sensitive.

The following officers were elected: Dr. P. H. Wright, Senatobia, President; Dr. J. P. Broadstreet, Grenada, Vice President; Dr. H. T. Stewart, Greenville, Secretary; Dr. C. C. Crowder, Kosciusko, Treasurer.

Dr. R. K. Luckie read an impressive eulogy in memory of the late Dr. W. L. Stovall.

Adjourned to meet in Jackson the first Wednesday in April, 1898.

